# The effect of Er^3+^ concentration on the kinetics of multiband upconversion in NaYF_4_:Yb/Er microcrystals

**DOI:** 10.3389/fchem.2023.1097250

**Published:** 2023-01-20

**Authors:** Hanchang Huang, Yanyi Zhong, Mingchen Li, Wenda Cui, Tongcheng Yu, Guomin Zhao, Zhongyang Xing, Chuan Guo, Kai Han

**Affiliations:** ^1^ College of Advanced Interdisciplinary Studies, National University of Defense Technology, Changsha, China; ^2^ Nanhu Laser Laboratory, National University of Defense Technology, Changsha, China; ^3^ State Key Laboratory of Pulsed Power Laser Technology, Changsha, China

**Keywords:** kinetics, highly doping Er, microcrystal, multiband upconversion, NaYF_4_

## Abstract

In Yb-Er co-doped upconversion (UC) nanomaterials, upconversion luminescence (UCL) can be modulated to generate multiband UCL emissions by changing the concentration of activator Er^3+^. Nonetheless, the effect of the Er^3+^ concentrations on the kinetics of these emissions is still unknown. We here study the single *β*-NaYF_4_:Yb^3+^/Er^3+^ microcrystal (MC) doped with different Er^3+^ concentrations by nanosecond time-resolved spectroscopy. Interestingly, different Er^3+^ doping concentrations exhibit different UCL emission bands and UCL response rates. At low Er^3+^ doping concentrations (1 mol%), multiband emission in *β*-NaYF_4_:Yb^3+^/Er^3+^ (20/1 mol%) MCs could not be observed and the response rate of UCL was slow (5–10 μs) in *β*-NaYF_4_:Yb^3+^/Er^3+^. Increasing the Er^3+^ doping concentration to 10 mol% can shorten the distance between Yb^3+^ ions and Er^3+^ ions, which promotes the energy transfer between them. *β*-NaYF_4_:Yb^3+^/Er^3+^ (20/10 mol%) can achieve obvious multiband UCL and a quick response rate (0.3 µs). However, a further increase in the Er doping concentration (80 mol%) makes MCs limited by the CR process and cannot achieve the four-photon UC process (^4^F_5/2_ → ^2^K_13/2_ and ^2^H_9/2_ → ^2^D_5/2_). Therefore, the result shows that changing the Er^3+^ doping concentration could control the energy flow between the different energy levels in Er^3+^, which could affect the response time and UCL emission of the Yb/Er doped rare earth materials. Our work can facilitate the development of fast-response optoelectronics, optical-sensing, and display industries.

## 1 Introduction

Lanthanide-doped nanomaterials have been widely studied in optics and biology due to luminescence multi-modulation at the micro-nano scale. Lanthanide-based upconversion (UC) microcrystals (MCs) transform infrared photons with low energy into high-energy photons (ultraviolet or visible), and have thus been increasingly used in laser (Chen et al., 2016), photovoltaics (Liang et al., 2013), storage (Zheng et al., 2018), and anticounterfeiting (Li et al., 2022). *β*-NaYF_4_ is the most efficient UC material lattice because of low phonon energy. Co-doping typical sensitizer Yb^3+^ and activator Er^3+^ in NaYF_4_ achieves multi-photons UC and exhibits multiband emission under saturated excitation (Yuan et al., 2018; Frenzel et al., 2021); this has potential application in super-resolution (Liu et al., 2017), optical multiplexing (Lu et al., 2013), and display (Zhang et al., 2015; Gao et al., 2020). Recent studies have demonstrated that the luminescence properties of upconversion nanoparticles (UCNPs) can be effectively enhanced (Han et al., 2014) or modulated by changing the structure (Wang et al., 2014), doping concentration (Wen et al., 2018), and surface modification (Zhou B et al., 2020), thus extending the application of UCNPs. In particular, the increasing study of highly doped Er^3+^ ions has shown that a high degree of energy migration between them occurs to suppress the effect of concentration quenching upon surface coating (Chen et al., 2017). The self-sensitization of Er ions has also been reported to directly achieve the modulation of energy transfer within UCNPs (Zhou J et al., 2020). These suggest that highly doped UCNPs are important for luminescence modulation.

High irradiation power density conditions allow the highly doped *β*-NaYF_4_:Yb^3+^/Er^3+^ MCs to exhibit stronger emissions. Huang *et al.* achieved nearly white luminescence in a single *β*-NaYbF_4_: Er^3+^ (2 mol%) MC; they studied the effect of Yb^3+^ ion doping concentration on UC kinetics (Huang et al., 2022b). For the UC system, the Yb^3+^ ion is the absorption antenna of the photon, and the Er^3+^ ion is the luminescence center of the MCs. The doping concentration of the Er^3+^ ion will directly affect the luminescence characteristics of the MCs. However, the effect of Er^3+^ concentration on UC kinetics still seems unknown. Moreover, an advanced setup is needed to better understand the kinetics of *β*-NaYF_4_:Yb^3+^/Er^3+^ systems with different Er^3+^ doping concentrations at nanosecond timescale. Optical trapping time-resolved photoluminescence spectroscopy (OT-TRPLS) combines a nanosecond pulsed laser (976 nm), an optical tweezer (with 1342 nm laser), and an advanced time-resolved photoluminescence device (Huang et al., 2022a). It has the ability to measure the nanosecond timescale transient spectra by trapping a single MC to deter the effect resulting from the MC movement and its interactions with other surrounding MCs.

In this paper, we investigate the effect of Er^3+^ concentration on the multiband emissions in the *β*-NaYF_4_:Yb^3+^/Er^3+^ MCs which are optically trapped by our OT-TRPLS platform. The kinetics were quantificationally resolved on the basis of the time-resolved spectra. Interestingly, under the 20 mol% condition of Yb^3+^ doping concentration, different Er^3+^ doping concentrations modulated MCs to exhibit different kinetic processes and response rates of upconversion luminescence (UCL). NaYF_4_:Yb^3+^/Er^3+^ (20/10 mol%) populated the characteristic ^2^P_9/2_ energy level of Er^3+^ ion (the energy level corresponding to a four-photon absorption) as fast as ∼0.1 μs after the excitation. In contrast, *β*-NaYF_4_:Yb^3+^/Er^3+^ (20/1 mol%) needed a timescale of 5–10 m after the excitation. However, further increasing the doping Er^3+^ concentration in *β*-NaYF_4_:Yb^3+^/Er^3+^ MCs, MCs cannot achieve the four-photon UC process. The time-evolution spectra of NaYF_4_:Er 10 and 80 mol% MCs show that single doping Er^3+^ ions cannot significantly improve the response rate of the UCL due to the smaller absorption cross section of Er^3+^ near 976 nm. The result indicates that increasing the doping concentration of Er^3+^ in *β*-NaYF_4_:Yb^3+^/Er^3+^ shortens the distance between Yb^3+^ and Er^3+^, thus changing the rate at which Yb ions transfer energy to Er ions. In addition, the cross-relaxation (CR) of highly doping Er ions will make the MCs unable to achieve the four-photon UC process.

## 2 Experimental

### 2.1 Materials

Yb^3+^, Er^3+^, and Y^3+^ were provided by ytterbium (III) chloride (YbCl_3_‧6H_2_O, 99.99%), erbium (III) chloride (ErCl_3_‧6H_2_O, 99.99%), and yttrium (III) chloride (YCl_3_‧6H_2_O, 99.99%) from Aladdin Industrial Corporation. Sodium fluoride (NaF), Na_2_-ethylenedia-metracetic acid (EDTA-2Na), and other chemical reagents were purchased from Sinopharm Chemical Reagent Co., Ltd.

### 2.2 Preparation of *β*-NaYF_4_:Yb^3+^/Er^3+^ MCs

NaYF_4_:Yb^3+^/Er^3+^ MCs were prepared by the hydrothermal method. To ensure a total rare-earth-ion content of exactly 1 mmol, YbCl_3_‧6H_2_O, ErCl_3_‧6H_2_O, and YCl_3_‧6H_2_O of different qualities from the specific doping ratio were dissolved in the deionized water (22 mL). Thereafter, ultrasonic stirring occurred until the solution became transparent; 1 mmol EDTA-2Na was then added and stirred into the mixed solution (30 min). Then, 10 mmol NaF was added and stirred for another 30 min until the solution became colloidal. Subsequently, the mixtures were transferred to a hydrothermal reactor for 24 h annealing at 160°C and then cooled. The final product was thrice washed with ethanol and deionized water.

### 2.3 Sample characterization

**FIGURE 1:**
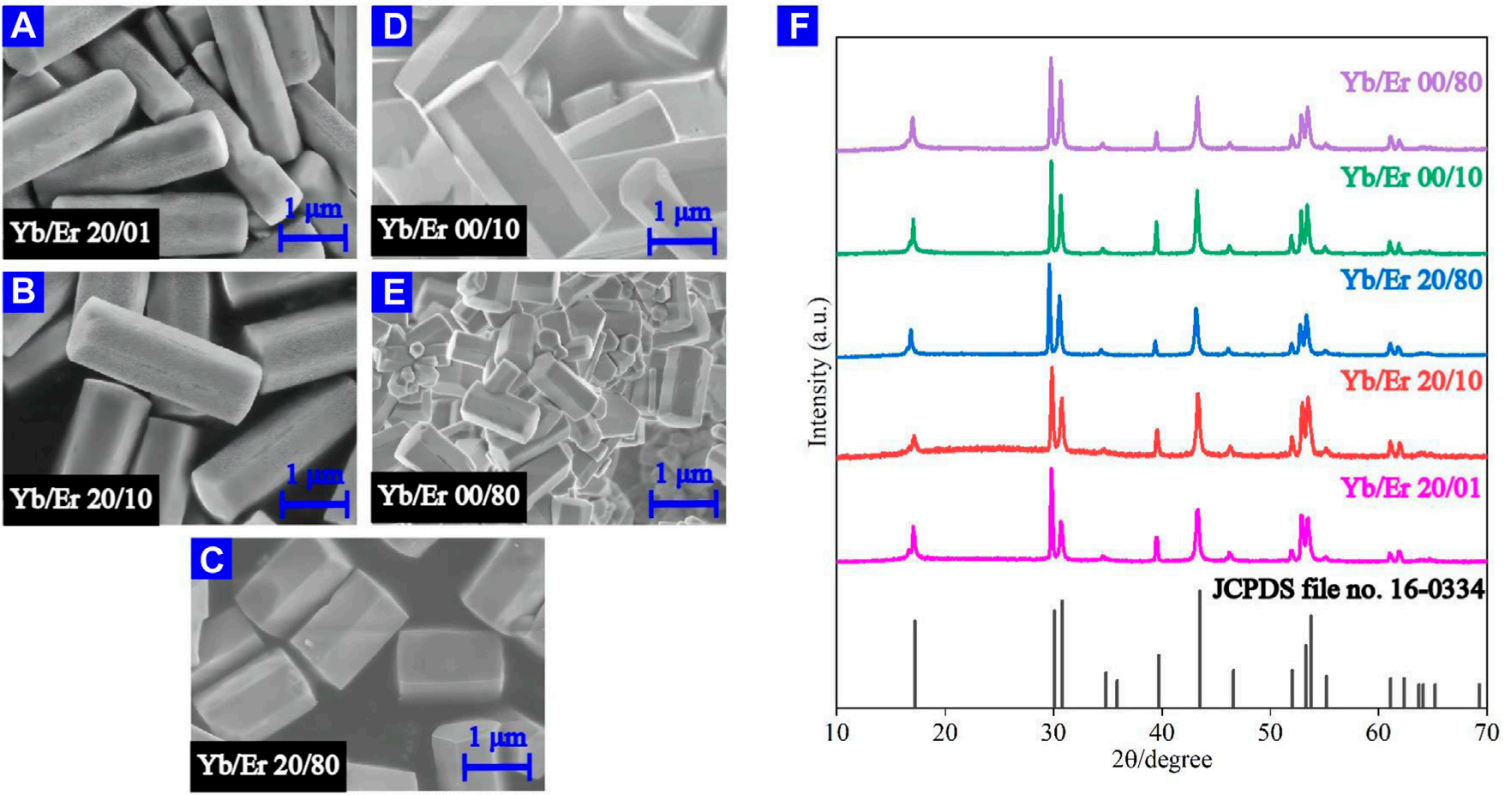
**(A–E)** SEM micrographs of *β*-NaYF_4_:Yb/Er MCs. **(F)** XRD patterns of the measured *β*-NaYF_4_:Yb^3+^/Er^3+^ MCs compared to the standard JCPDS file no.16-0334.

NaYF_4_:Yb^3+^/Er^3+^ MCs of the *β* crystalline phase were characterized by scanning electron microscopy (SEM). The hexagonal MCs were about 2 μm in length (Figures 1A–E). An X-ray diffractometer with Cu K radiation was employed at 40 kV and 200 mA (Rigaku) to record the X-ray diffraction (XRD) patterns of the MCs. The XRD data in the 2θ range from 10° to 70° were collected at a 10° min^−1^ scanning rate. Based on the Joint Committee on Powder Diffraction Standards (JCPDS), it was demonstrated that the prepared NaYF_4_ MCs are *β*-phase crystalline (Figure 1F).

### 2.4 Photoluminescence measurements

**FIGURE 2:**
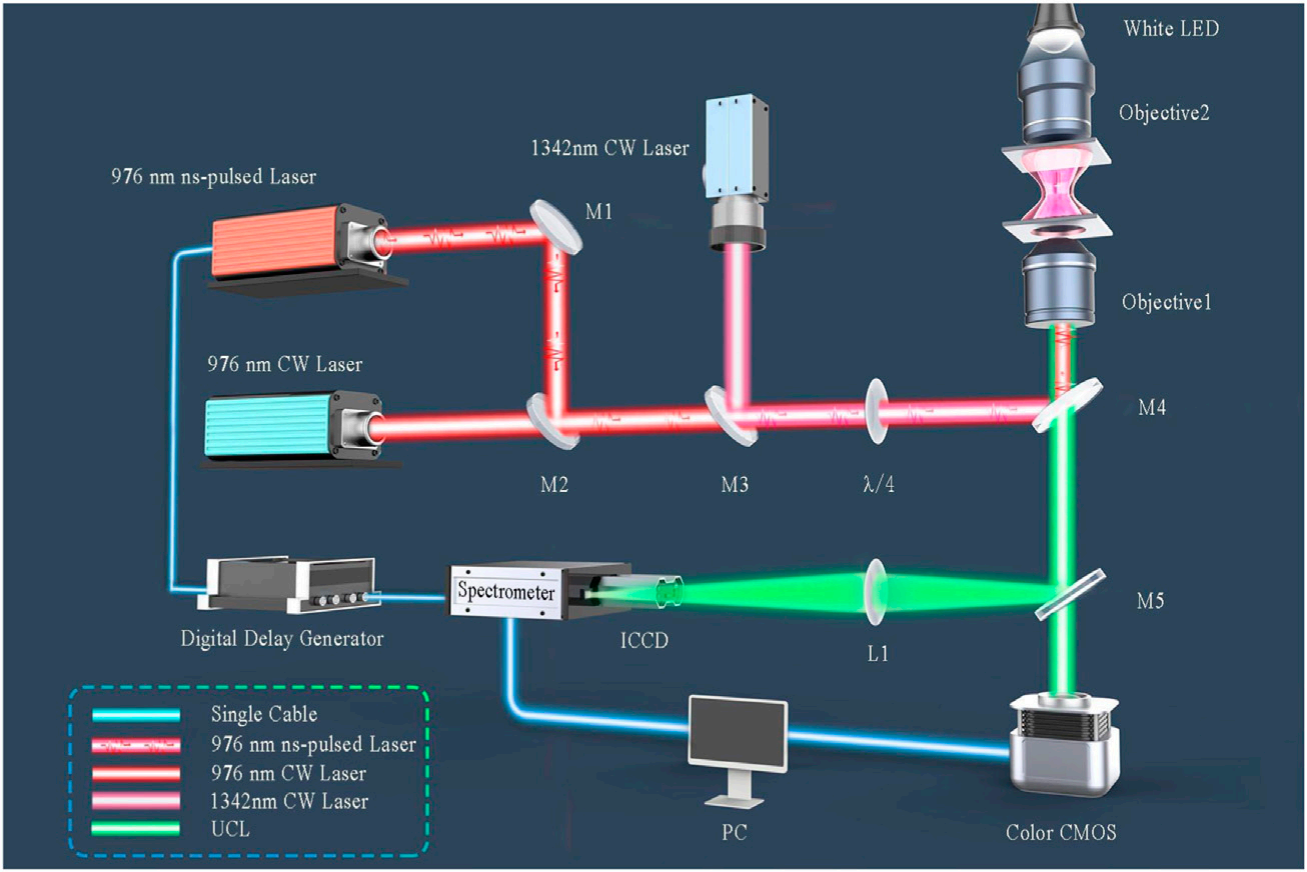
Setups for UCL detection with the self-built OT-TRPLS device. M1, planar reflective silver mirror; M2, polarization beam-combining mirror (for a 976 nm laser); M3, beam-combining mirror (for a 976 nm laser and 1342 nm laser); M4, dichroic mirror for short-wave pass and the cutoff wavelength is 900 nm; M5, 50:50 beam-splitter. Objective 1 is an oil lens (1.3 NA, 100x). Objective 2 has 10 × magnification with .25 NA for lighting focusing. All lasers were coaxial.

We used OT-TRPLS to measure the time evolution spectra. As previously mentioned, optical tweezers (OT) ensured the stability and isolation of the sample as well as avoiding any undesired interaction by the sample with its surroundings. The 1342 nm excitation laser works as OT for the low absorption coefficients of both Er^3+^ and Yb^3+^ ions at this wavelength. The optical trapping process was observed using a color-complementary metal oxide semiconductor (CMOS). The peak excitation power density was ∼0.32 GW cm^−2^, and the pulse width of the laser was 15 ns. The time evolution spectra were captured using an ICCD camera. A 976 nm ns-pulsed laser source was utilized in OT-TRPLS under the control of digital delay generator (DDG) at a repetition rate of 37 Hz (see Figure 2).

## 3 Results and discussion

**FIGURE 3:**
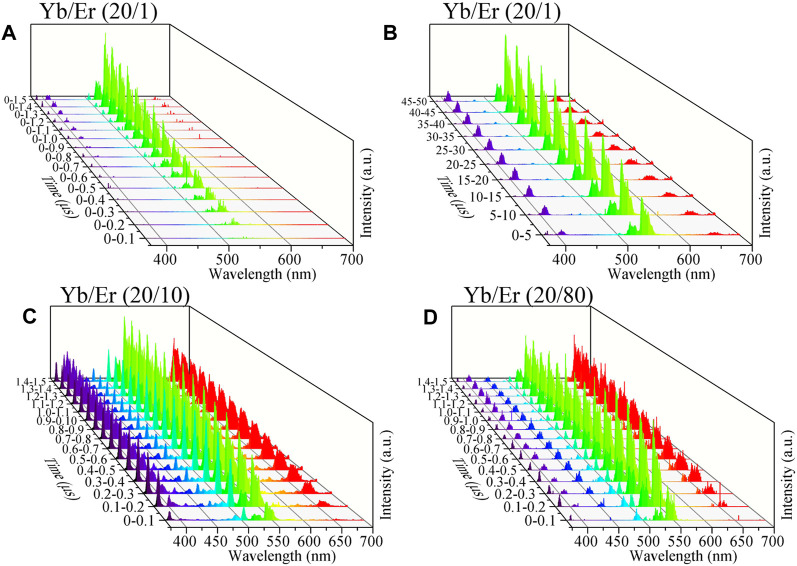
NaYF_4_:Yb^3+^/Er^3+^ evolution spectrum of Yb^3+^/Er^3+^(20/x mol%) under 976 nm pulse excitation. **(A, B)** *x* = 1, **(C)** *x* = 10, and **(D)** *x* = 80. The 976 nm ns-pulsed excitation starts from the 0 moment.

We captured the time evolution spectra of optically trapped NaYF_4_:Yb^3+^/Er^3+^ MCs after the 976 nm ns-pulsed laser excitation (see Figure 3). Our results indicate that NaYF_4_:Yb^3+^/Er^3+^ MCs show sequent UCL emissions at different wavelengths and that different Er^3+^ doping concentrations can modulate UCL emissions. The main emissions are 522 nm, 542 nm, 410 nm, 558 nm, and 654 nm for NaYF_4_:Yb^3+^/Er^3+^ (20/1 mol%) MCs within 1 μs after excitation (see Figure 3A). In the timescale from 5 to 10 µs, NaYF_4_:Yb^3+^/Er^3+^ (20/1 mol%) MCs show weak 402 nm and 470 nm emissions (see Figure 3A). Increasing the Er^3+^ doping concentration to 10 mol%, NaYF_4_:Yb^3+^/Er^3+^ (20/10 mol%) MCs show multiband UCL emissions within 0.3 µs, such as 522 nm, 542 nm, 410 nm, 558 nm, 654 nm, 505 nm, 384 nm, 496 nm, 430 nm, 482 nm, 617 nm, 654 nm, 456 nm, 443 nm, 575 nm, 585 nm, and 640 nm. However, when the concentration of Er^3+^ ions is increased to 80 mol%, NaYF_4_:Yb^3+^/Er^3+^ (20/80 mol%) MCs only show partly multiband UCL emissions. The concentration of doping Er^3+^ could effectively change the UCL emission under 976 nm ns-pulsed excitation.

**FIGURE 4:**
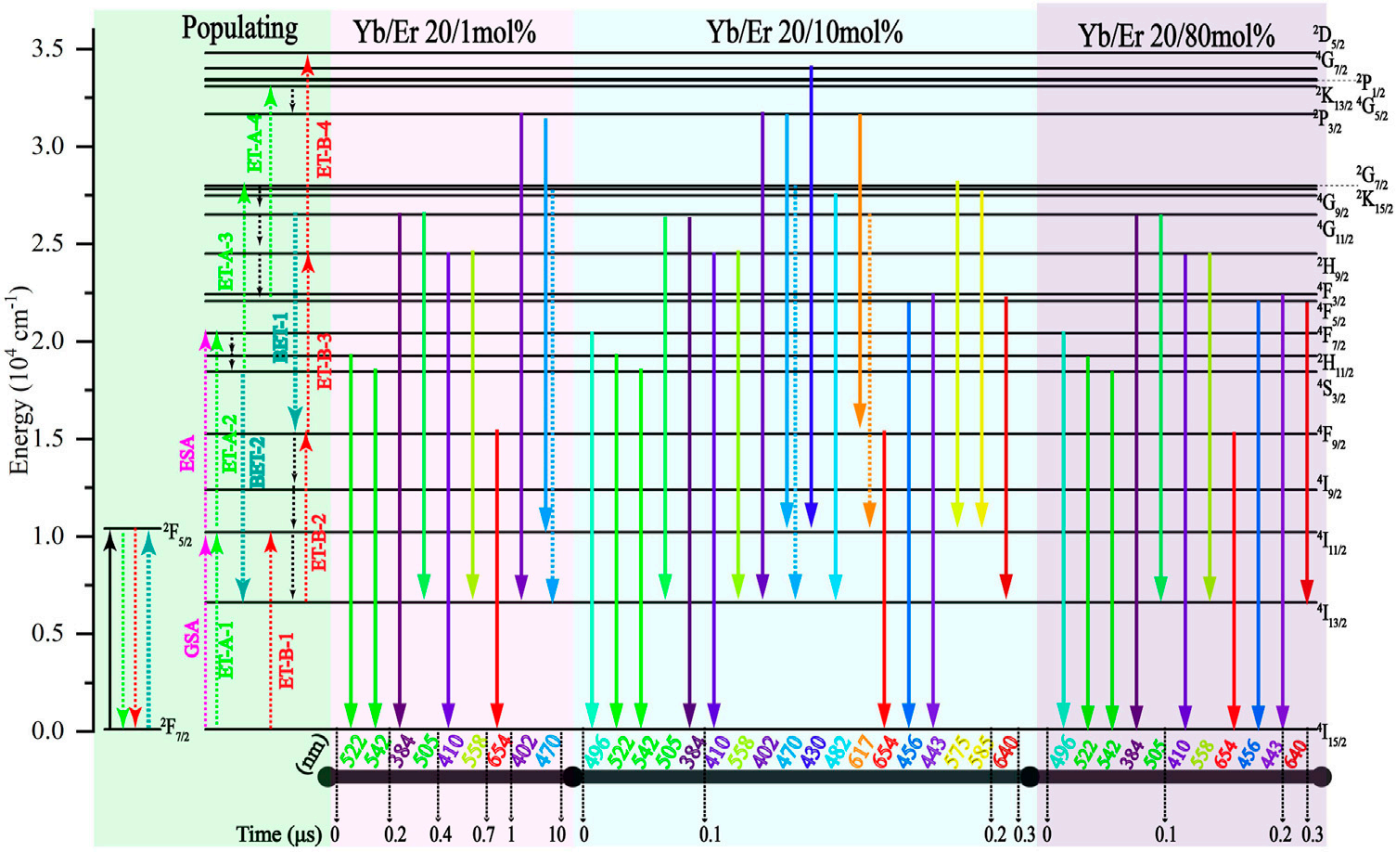
Transition mechanism of NaYF_4_:Yb^3+^/Er^3+^ (20/x mol%) with different Er^3+^ doping concentrations under 976 nm pulse excitation. The 976 nm ns-pulsed excitation starts from the 0 moment. Green part is the populating process. Red part is the radiative transition process at *x* = 1. Blue part is the radiative transition process at *x* = 10. Purple part is the radiative transition process at *x* = 80.

Next, we focused on the major UCL peaks; previous studies (Cheng et al., 2002; Sardar et al., 2003; Wegh et al., 2003; Chen et al., 2007; O'Shea et al., 2007; Yuan et al., 2018; Frenzel et al., 2021) indicate energy levels of Er^3+^ ions and their corresponding emission wavelengths (Figure 4. Pathway A (^4^I_15/2_ → ^4^I_11/2_ → ^4^F_7/2_ → ^2^H_11/2_ →^4^S_3/2_ → ^2^G_7/2_ → ^4^G_11/2_ → ^2^H_9/2_ → ^4^F_5/2_ → ^2^K_13/2_) and pathway B (^4^I_15/2_ → ^4^I_11/2_ → ^4^I_13/2_ → ^4^F_9/2_ → ^2^H_9/2_ → ^2^D_5/2_) are two main channels where energy levels are populated to achieve UCL emission. (Anderson et al., 2014; Jung et al., 2015; Yuan et al., 2018; Frenzel et al., 2021).

For NaYF_4_:Yb^3+^/Er^3+^ (20/1 mol%), the UCL at 542 nm (^4^S_3/2_→^4^I_15/2_) and 522 nm (^2^H_11/2_→^4^I_15/2_) emits within 0.2 μs of the 976 nm ns-pulsed excitation. The aforementioned emissions originated from ^4^I_15/2_ → ^4^I_11/2_ → ^4^F_7/2_ → ^2^H_11/2_ →^4^S_3/2_ of pathway A. In the timescale from 0.2 μs to 0.4 μs after excitation, the emergence of 505 nm (^4^G_11/2_ → ^4^I_13/2_) and 384 nm (^4^G_11/2_ → ^4^I_15/2_) demonstrates that ^4^G_11/2_ has accumulated electron population by ^4^I_15/2_ → ^4^I_11/2_ → ^4^F_7/2_ → ^2^H_11/2_ →^4^S_3/2_ → ^2^G_7/2_ → ^4^G_11/2_ from pathway A. Then, in the timescale from 0.4 μs to 0.7 μs after excitation, the emergence of 410 nm (^2^H_9/2_ → ^4^I_15/2_) and 558 nm (^2^H_9/2_ → ^4^I_13/2_) demonstrates that ^2^H_9/2_ has accumulated an electron population by ^4^G_11/2_ → ^2^H_9/2_. Although the ^2^H_9/2_ energy level could be populated by ^4^I_15/2_ → ^4^I_11/2_ → ^4^I_13/2_ → ^4^F_9/2_ → ^2^H_9/2_ of pathway B, no obvious electron population accumulated at the ^4^F_9/2_ energy level. The 654 nm (^4^F_9/2_ → ^4^I_15/2_) is not observed at this time. In the timescale from 0.7 μs to 1 μs after excitation, the emergency of 654 nm emission indicates that an ^4^F_9/2_ energy level has accumulated an electron population. However, in the timescale from 5 μs to 10 μs after excitation, the emergence of 402 nm (^2^P_3/2_ → ^4^I_13/2_) and 470 nm (^2^P_3/2_ → ^4^I_11/2_) emissions demonstrates the occurrence of the four-photon process from pathway A (^4^I_15/2_ → ^4^I_11/2_ → ^4^F_7/2_ → ^2^H_11/2_ →^4^S_3/2_ → ^2^G_7/2_ → ^4^G_11/2_ → ^2^H_9/2_ → ^4^F_5/2_ → ^2^K_13/2_). The four-photon process from pathway B is not observed by UCL, such as 430 nm (^4^G_7/2_ → ^4^I_11/2_). It can be concluded through data and analysis that pathway A is more effective than B in NaYF_4_:Yb^3+^/Er^3+^ (20/1 mol%).

For NaYF_4_:Yb^3+^/Er^3+^ (20/10 mol%), the UCL at 542 nm (^4^S_3/2_→^4^I_15/2_), 522 nm (^2^H_11/2_→^4^I_15/2_), 496 nm (^4^F_7/2_ → ^4^I_15/2_), 505 nm (^4^G_11/2_ → ^4^I_13/2_), and 384 nm (^4^G_11/2_ → ^4^I_15/2_) emits within 0.1 μs after 976 nm ns-pulsed excitation, which originated from pathway A. In the timescale from 0.1 μs to 0.2 μs after excitation, the emergence of 410 nm (^2^H_9/2_ → ^4^I_15/2_) 558 nm (^2^H_9/2_ → ^4^I_13/2_), 402 nm (^2^P_3/2_ → ^4^I_13/2_), 470 nm (^2^P_3/2_ → ^4^I_11/2_) 654 nm (^4^F_9/2_ → ^4^I_15/2_), and 430 nm (^4^G_7/2_ → ^4^I_11/2_) demonstrates that ^4^F_9/2_, ^4^G_11/2_, ^2^H_9/2_, ^2^P_3/2_, and ^4^G_7/2_ energy levels have accumulated electron population by four-photon pathway A and four-photon pathway B. Moreover, 482 nm (^2^K_15/2_ → ^4^I_13/2_), 575 nm (^2^G_7/2_ → ^4^I_11/2_), 585 nm (^4^G_9/2_ → ^4^I_11/2_), 456 nm (^4^F_5/2_ → ^4^I_15/2_), and 443 nm (^4^F_3/2_ → ^4^I_15/2_) emissions indicate that the ^2^K_15/2_, ^2^G_7/2_, ^4^G_9/2_, ^4^F_3/2_, and ^4^F_5/2_ levels can accumulate electron populations. Compared with NaYF_4_:Yb^3+^/Er^3+^ (20/1 mol%) MCs, NaYF_4_:Yb^3+^/Er^3+^ (20/10 mol%) exhibited more UCL emissions and a faster response rate of UCL. This indicates that increasing the Er^3+^ ion-doping concentration can effectively enhance the UC process of NaYF_4_:Yb^3+^/Er^3+^ MCs in specific concentration ranges of Er^3+^.

Further increasing the doping concentration of Er^3+^, for NaYF_4_:Yb^3+^/Er^3+^ (20/80 mol%), the UCL at 542 nm (^4^S_3/2_→^4^I_15/2_), 522 nm (^2^H_11/2_→^4^I_15/2_), 496 nm (^4^F_7/2_ → ^4^I_15/2_), 505 nm (^4^G_11/2_ → ^4^I_13/2_), and 384 nm (^4^G_11/2_ → ^4^I_15/2_) emits within .1 μs after 976 nm ns-pulsed excitation, which originated from pathway A. However, in the timescale from .1 μs to .2 μs after excitation, only 410 nm (^2^H_9/2_ → ^4^I_15/2_), 558 nm (^2^H_9/2_ → ^4^I_13/2_), 654 nm (^4^F_9/2_ → ^4^I_15/2_), 456 nm (^4^F_5/2_ → ^4^I_15/2_), and 443 nm (^4^F_3/2_ → ^4^I_15/2_) emissions could be observed, demonstrating that ^2^H_9/2_, ^4^F_9/2_, ^4^F_5/2_, and ^4^F_3/2_ energy levels have accumulated electron populations. The UCL emissions from four-photon pathways are not observed. This indicates that further increasing the Er^3+^ doping concentration can increase the response rate of the UCL, but the kinds of emissions are reduced and the UCL emissions from the four-photon UC process could not emit. For NaYF_4_:Yb/Er (20/X mol%) MCs, 80 mol% Er doping concentration takes 0.2 μs to achieve the A-1, A-2, A-3, B-1, B-2, and B-3 ET processes, while 1 mol% Er doping concentration takes 1 μs. This indicates that for NaYF_4_:Yb/Er (20/80 mol%) MCs, the two- and three-photon UCLs maintain a high response rate even if all Y^3+^ is replaced with Er^3+^. However, for high Er^3+^ ion doping concentration (80 mol%), the CR processes of Er^3+^ prevents the energy levels of the four-photon process from accumulating effective populations, thus preventing the emission of typical four-photon UCLs such as 402 and 430 nm.

**FIGURE 5:**
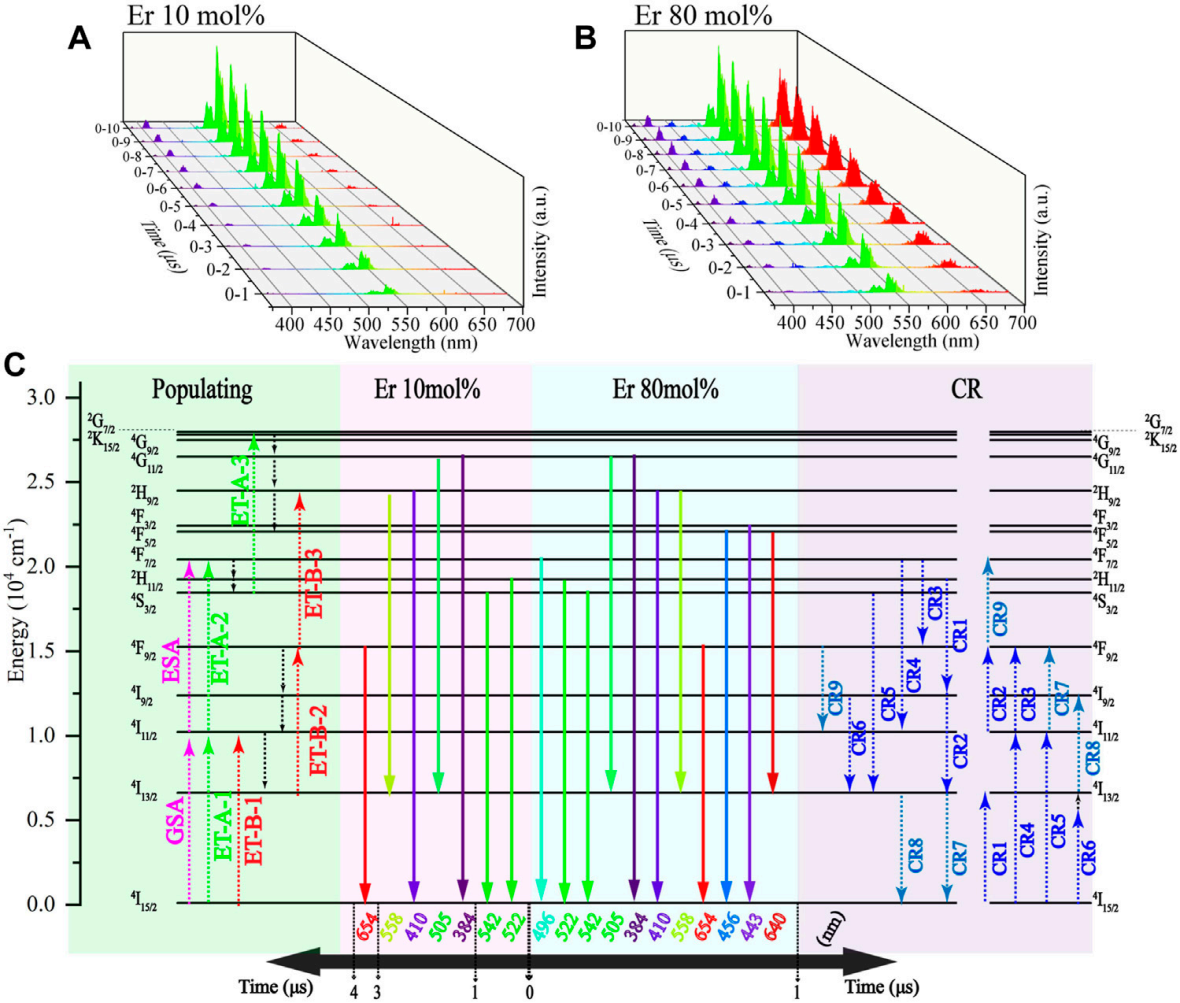
NaYF_4_:Er^3+^ evolution spectrum under 976 nm pulse excitation: **(A)** Er 10 mol% and **(B)** Er 80 mol%. **(C)** Schematic representation of the transition kinetics in NaYF_4_:Yb^3+^/Er^3+^ (00/x mol%) with different Er^3+^ doping concentrations under 976 nm pulse excitation. Green part is the populating process. Red part is the radiative transition process at *x* = 10. Blue part is the radiative transition process at *x* = 80. Purple part is the CR process. The 976 nm ns-pulsed excitation starts from the 0 moment.

To further study the influence of Er^3+^ on the kinetics of multiband UCL, we measured and analyzed the time evolution spectra of NaYF_4_:Er^3+^ MCs with different Er^3+^ doping concentrations (Figures 5A, B. According to the result of the evolution spectrum, both NaYF_4_:Er^3+^ (10 mol%) and NaYF_4_:Er^3+^ (80 mol%) can achieve the UC process by Er^3+^ ion self-sensitization. Nonetheless, without Yb^3+^ ion sensitization, the UCL of NaYF_4_:Er^3+^ MCs was weakened and the UCL response rate slowed. According to the time evolution spectrum and a previous report on Er^3+^ self-sensitization (Lu et al., 2014), the schematics of the transition kinetics are presented in Figure 5C. NaYF_4_:Er^3+^ (10 mol%) cannot emit UCL from a four-photon ET process. Moreover, the UCL response rate is relatively slow, and it takes 4 μs to perform three-photon pathways A and B. Increasing Er^3+^ doping concentration in NaYF_4_, NaYF_4_:Er^3+^ (80 mol%) could absorb more photons by the self-sensitization of Er^3+^ ions. However, NaYF_4_:Er^3+^ (80 mol%) cannot emit UCL by a four-photon UC process. In addition, the UCL response rate in NaYF_4_:Er^3+^ (80 mol%) is at the timescale of ∼1 μs for achieving three-photon pathways A and B. In contrast, for NaYF_4_:Yb^3+^/Er^3+^ (20/80 mol%) and NaYF_4_:Yb^3+^/Er^3+^ (20/10 mol%), the UCL response rates are at the timescale of ∼0.2 μs to achieve three-photon pathways A and B. This suggests that the self-sensitization of Er^3+^ is not the main factor for the changing response rate in NaYF_4_:Yb^3+^/Er^3+^.

**FIGURE 6:**
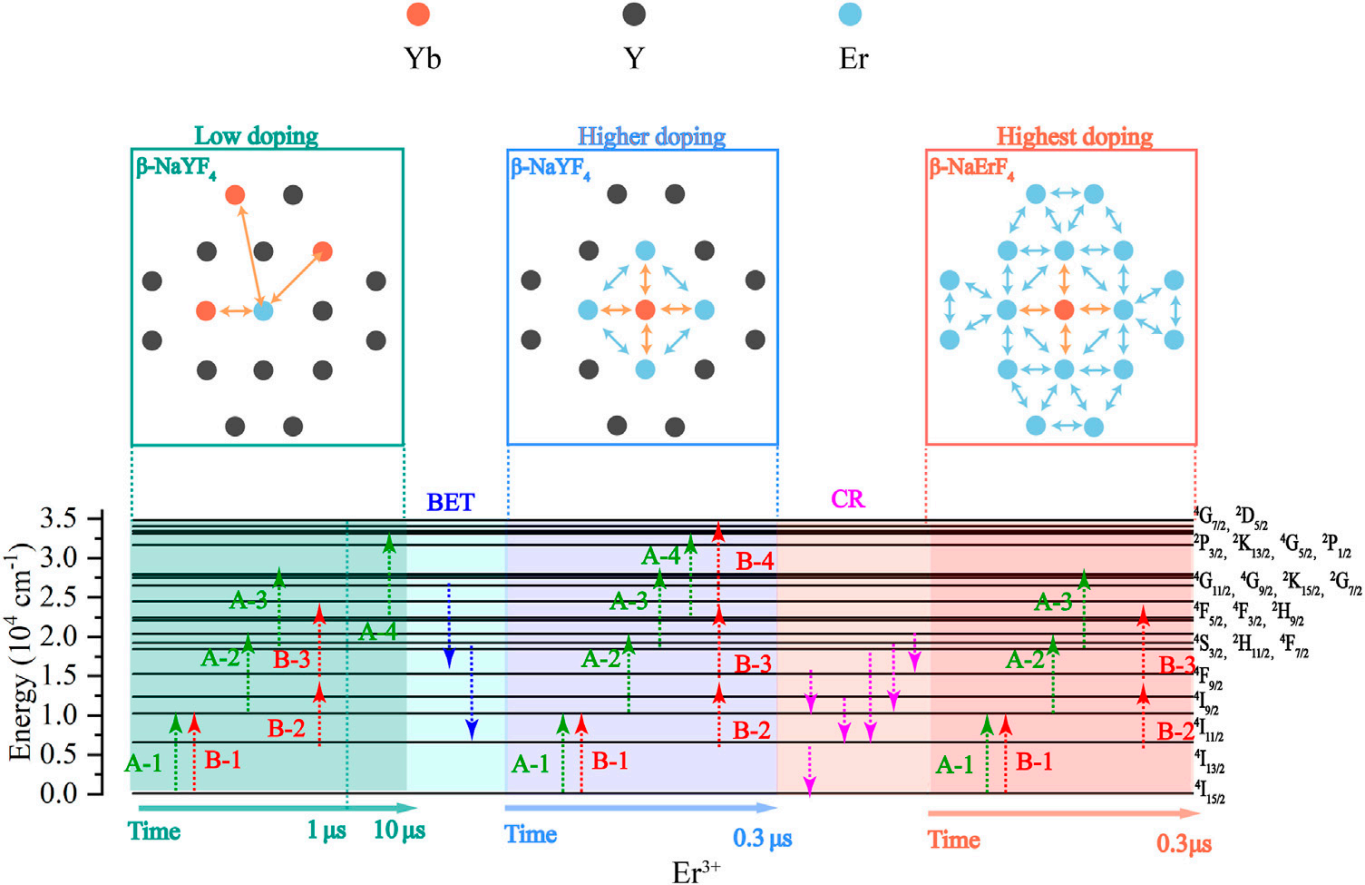
Schematic diagram of the influence of Er^3+^ doping concentration on the UCL emission of NaYF_4_:Yb^3+^/Er^3+^ MCs under the excitation of 976 nm laser.

More interestingly, in studying the influence of the Er^3+^ doping concentration on the distribution of doped ions in NaYF_4_:Yb^3+^/Er^3+^ (20/x mol%) MCs (see Figure 6), increasing the Er^3+^ doping concentration can significantly shorten the distance between Yb^3+^ ions and Er^3+^ ions, which could improve the energy transfer between them (Dexter, 1953). When Er^3+^ doping concentration is low, such as NaYF_4_:Yb^3+^/Er^3+^ (20/1 mol%), the distance between Yb^3+^ ions and Er ions is great. Yb^3+^ ions transfer little energy to Er^3+^, leading to a slow UCL response rate of MCs; these can only show part of the four-photon UC process (A-4). Increasing the concentration of Er^3+^ doping can shorten the distance between Yb^3+^ and Er^3+^ ions such as NaYF_4_:Yb^3+^/Er^3+^ (20/10 mol%) MCs, which facilitate the energy transfer between Yb^3+^ ions and Er^3+^ ions. Hence, NaYF_4_:Yb^3+^/Er^3+^ (20/10 mol%) MCs show obvious multiband UCL and a fast UCL response rate. When further increasing the Er^3+^ doping concentration, such as NaYF_4_:Yb^3+^/Er^3+^ (20/80 mol%), the complex CR process reduces the electron population of Er^3+^, and NaYF_4_:Yb^3+^/Er^3+^ (20/80 mol%) cannot achieve a four-photon UC process. However, the shorter Yb^3+^–Er^3+^ distance makes the NaYF_4_:Yb^3+^/Er^3+^ (20/80 mol%) UCL response rate faster.

## 4 Conclusion

In summary, we utilized the OT-TRPLS to trap single *β*-NaYF_4_:Yb^3+^/Er^3+^ MC varying Er^3+^ activator doping concentration and measured the time evolution spectrum. The result shows that varying the Er^3+^ doping concentration can change the UCL emitting waveband and response rate, which indicates different UC kinetics. The time-evolution spectra of *β*-NaYF_4_:Yb^3+^/Er^3+^ MCs by single-doped Er show that the self-sensitization of Er^3+^ ions is not a major factor in the change of the UCL kinetics of MCs. The varying Er^3+^ doping concentration changes the Yb^3+^–Er^3+^ distance, which leads to a different energy transfer between Er and Yb ions. An appropriate increase in the Er^3+^ doping concentration can promote the multiband emission and UCL response rate under strong ns-pulse excitation, such as NaYF_4_:Yb^3+^/Er^3+^ (20/1 mol%) and NaYF_4_:Yb^3+^/Er^3+^ (20/10 mol%). However, the CR process inhibits the population of electrons at the higher energy levels of Er^3+^ ions, leading to the inability of the four-photon UC process to be realized in NaYF_4_:Yb^3+^/Er^3+^ (20/80 mol%) MCs. Our research offers a new understanding of the influence of Er^3+^ concentration on UC, which is promising in fast-response optoelectronics, optical-sensing, and display industries.

## Data Availability

The original contributions presented in the study are included in the article/Supplementary Material; further inquiries can be directed to the corresponding authors.
